# Using the synthetic form RS5 to obtain new introgressive lines of common wheat

**DOI:** 10.18699/VJ21.088

**Published:** 2021-11

**Authors:** R.O. Davoyan, I.V. Bebyakina, E.R. Davoyan, Y.S. Zubanova, D.M. Boldakov, D.S. Mikov, V.A. Bibishev, A.N. Zinchenko, E.D. Badaeva

**Affiliations:** National Center of Grain named after P.P. Lukyanenko, Krasnodar, Russia; National Center of Grain named after P.P. Lukyanenko, Krasnodar, Russia; National Center of Grain named after P.P. Lukyanenko, Krasnodar, Russia; National Center of Grain named after P.P. Lukyanenko, Krasnodar, Russia; National Center of Grain named after P.P. Lukyanenko, Krasnodar, Russia; National Center of Grain named after P.P. Lukyanenko, Krasnodar, Russia; National Center of Grain named after P.P. Lukyanenko, Krasnodar, Russia; National Center of Grain named after P.P. Lukyanenko, Krasnodar, Russia; Vavilov Institute of General Genetics of the Russian Academy of Sciences, Moscow, Russia

**Keywords:** common wheat, synthetic forms, disease resistance, protein, cytological analysis, C-banding, substituted chromosomes, translocations, gluten, мягкая пшеница, синтетические формы, устойчивость к болезням, белок, клейковина, цитологический анализ, C-banding, замещенные хромосомы, транслокации

## Abstract

The use of the gene pool of wild relatives, which have a signif icant reserve of genetic diversity, is of immediate interest for breeding common wheat. The creation and use of synthetic forms as “bridges” is an effective method of transferring valuable genetic material from wild relatives to cultivated wheat. For this purpose, genome addition, genome substitution and recombinant “secondary” synthetic forms have been created in the P.P. Lukyanenko National Center of Grain. The synthetic recombination form RS5 (BBAASDt), in which the third genome consists of chromosomes of Aegilops speltoides (S) and Aegilops tauschii (Dt), was obtained from crossing the synthetic forms Avrodes (BBAASS) and M.it./ Ae. tauschii (BBAADt Dt), in which the D genome from Ae. tauschii was added to the BBAA genomes of the durum wheat cultivar Mutico italicum. Introgression lines resistant to leaf rust, yellow rust and powdery mildew have been obtained from backcrosses with the susceptible common wheat cultivars Krasnodarskaya 99, Rostislav and Zhirovka. Twelve resistant lines that additionally have high technological characteristics of grain and f lour have been selected. The cytological study (С-banding) has revealed chromosomal modif ications in 6 of 8 lines under study. The rearrangements mainly affected the chromosomes of the D genome, 1D, 3D, 4D, 6D and 7D. It was found that in most cases the genetic material from the synthetic form RS5 in the studied lines was represented by substituted chromosomes from Ae. tauschii. In line 5791p17, the substitution of chromosomes 6D from Ae. tauschii and 7D from Ae. speltoides was revealed. Substitutions 4D(4Dt), 6D(6Dt) from Ae. tauschii and 7D(7S) from Ae. speltoides were obtained for the f irst time. Molecular analysis of 12 lines did not reveal effective leaf rust resistance genes, presumably present in synthetic forms of M.it./Ae. tauschii and Avrodes. It is assumed that the lines may carry previously unidentif ied genes for fungal disease resistance, in particular for resistance to leaf rust, from Ae. tauschii and Ae. speltoides

## Abstract (ru)

Актуальной задачей селекции мягкой пшеницы является вовлечение генофонда диких сородичей, обладающих значительным запасом генетического разнообразия. Эффективный метод передачи ценного генетического материала от диких сородичей в культурную пшеницу – создание и использование в качестве «мостиков» синтетических форм. С этой целью в Национальном центре зерна им. П.П. Лукьяненко созданы геномно-замещенные, геномно-добавленные и рекомбинантные «вторичные» синтетические формы. Синтетическая форма RS5 (BBAASDt), у которой третий геном состоит из хромосом Aegilops speltoides (S) и Aegilops tauschii (Dt), была получена от скрещивания синтетических форм Авродес (BBAASS) и M.it./Ae. tauschii (BBAADtDt), у которой геном D от Ae. tauschii был добавлен к геномам BBAA твердой пшеницы Mutico italicum. От беккроссов с восприимчивыми к листовой ржавчине, желтой ржавчине и мучнистой росе сортами мягкой пшеницы Краснодарская 99, Ростислав и Жировка были получены устойчивые к этим болезням интрогрессивные линии. Отобраны 12 линий, которые наряду с устойчивостью к болезням имеют высокие технологические характеристики зерна и муки. Цитологический анализ (С-banding) выявил хромосомные перестройки у шести из восьми исследуемых линий. Перестройки в основном затронули хромосомы генома D – 1D, 3D, 4D, 6D и 7D. Установлено, что генетический материал от синтетической формы RS5 в изученных линиях в большинстве случаев представлен в виде замещенных хромосом от Ae. tauschii. В линии 5791п17 обнаружено замещение хромосом 6D от Ae. tauschii и 7D от Ae. speltoides. Хромосомные замещения 4D(4Dt), 6D(6Dt) от Ae. tauschii и 7D(7S) от Ae. speltoides получены впервые. Молекулярный анализ 12 линий не выявил у них эффективных генов устойчивости к листовой ржавчине, предположительно присутствующих в синтетических формах M.it./Ae. tauschii и Авродес. Сделано предположение, что линии могут нести не идентифицированные ранее гены устойчивости к грибным болезням, в частности к листовой ржавчине, от видов Ae. tauschii и Ae. speltoides.

## Introduction

Сommon wheat (Triticum aestivum L.) is one of the main food crops. The constantly growing need to increase its productivity against the background of global climate changes requires further intensification of the breeding process. One of the main conditions for this is the presence of sufficient genetic diversity and, in particular, disease resistance genes. An actual and effective way to expand the genetic diversity of common wheat is to use its numerous related wild and cultivated species as sources of valuable breeding traits (Rasheed et al., 2018). It should be noted that almost all effective diseases resistance genes of common wheat originate from the gene pool of its wild relatives (McIntosh et al., 2015).

One of the most effective methods of transferring valuable genetic material from wild relatives to common wheat is the creation and use of synthetic forms as “bridges”. An original approach was developed at the P.P. Lukyanenko National Center of Grain, which made it possible to create genome substituted, genome added and recombinant “secondary” synthetic forms (Zhirov, Ternovskaya, 1984; Davoyan R.O. et al., 2012). The genome substitution form of Avrodes (BBAASS) was used to create recombinant synthetic forms (RS-forms), in which, against the background of BA genomes, the third genome was recombinant and simultaneously consisted of two different wild species genomes (Davoyan E.R. et al., 2012). This form, due to the presence of the S genome from Ae. speltoides, has the ability to promote homoeologous pairing of chromosomes (Tsatsenco et al., 1993), which should have contributed to the production of new translocations and recombinations between chromosomes of different species.

The aim of the study was to use a synthetic form of RS5 (BBAASDt ), in which the third genome consists of Aegilops speltoides (S) and Ae. tauschii (Dt) chromosomes, to obtain new introgression lines of common wheat. This paper presents the results of cytological and molecular analysis, evaluation of resistance to fungal diseases, productivity components, technological qualities of grain and flour of common wheat introgression lines obtained using this synthetic form.

## Materials and methods

Introgression lines of common wheat (BC2F6–BC3F5) obtained with the participation of a synthetic form of RS5 made up the material for this study. Common wheat varieties Krasnodarskaya 99 (lines 4942p17, 5038p17, 5658p19, 5714p18, 5766p19, 5791p17, 5845p18), Rostislav (lines 5001p17, 5656p19) and Zhirovka (lines 5725p18, 5733p19, 5785p18), susceptible to leaf rust, yellow rust and powdery mildew, were used as recipient varieties. The Zhirovka variety has a translocation of 5BS.5BL-5GL, obtained from the species T. militinae through the synthetic form T. miguschovae. Translocation 1RS.1BL from rye was detected in variety Rostislav

The study of chromosome pairing in metaphase I of meiosis was carried out in maternal pollen cells on pressed preparations stained with acetic acid hematoxylin according to the generally accepted method (Pausheva, 1974). The number of cells studied in the lines ranged from 169 to 248.

The assessment of resistance to leaf and yellow rust was carried out at the stage of adult plants in the field, against the background of artificial infection. To assess the resistance to yellow rust, the Gassner and Straib scale was used (Gasner, Straib, 1934). Resistance to leaf rust was determined according to the Mains and Jakson scale (Mains, Jakson, 1926). Plants with reaction type 0 (immune), 1 (highly resistant) and 2 (moderately resistant) were classified as resistant. The resistance of plants with an intermediate type of reaction from 0 to 1 (single very small pustules with necrosis) was indicated by a score of 01. Plants with reaction type 3–4 were considered susceptible. Resistance to powdery mildew was evaluated on a natural infectious background according to the Geschele scale (Peresipkin, 1979). Plants with a degree of powdery mildew damage of 0–20 % were classified as resistant.

DNA extraction was carried out using the Plaschke et al. method (Plaschke et al., 1995). To identify the Lr genes, primers marking the Lr28, Lr35, Lr39 and Lr51 genes were used – CS421570-R, CS421570-L; BCD260F1, 35R2; GDM35-L, GDM35-R; S30-13L, AGA7-759R, respectively (Seyfarth et al., 1999; Singh et al., 2004; Cherukuri et al., 2005; Helguera et al., 2005). The PCR reaction was performed according to the conditions recommended by the authors. Electrophoresis of the PCR fragments was carried out similarly to those previously described (Davoyan E.R. et al., 2018).

Differential staining of chromosomes (C-banding) was performed at the Vavilov Institute of General Genetics according to the method developed by Badaeva and co-authors (Badaeva et al., 1994).

Technological quality of grain and flour was studied at the department of grain technology and biochemistry, P.P. Lukyanenko National Center of Grain, according to the Methods of State Crop Variety Trial (1988). Statistical processing of the obtained results was carried out using the AGROS-2.10 program.

## Results

The synthetic form RS5 showed high resistance to leaf and yellow rust and moderate resistance to powdery mildew, while having very low fertility. To transfer resistance and restore fertility, this form was crossed with susceptible to these diseases common wheat varieties Krasnodarskaya 99, Rostislav and Zhirovka. The first generation of hybrid plants was partially fertile and showed resistance to a complex of wheat diseases. Depending on the level of fertility of these plants, backcrossing with common wheat was performed from 1 to 3 times, but in most cases two backcrosses were sufficient to restore it. The plants obtained from backcrosses had from 40 to 42 chromosomes. The results of the cytological study of chromosomal associations in metaphase I of meiosis are shown in Table 1.

**Table 1.**
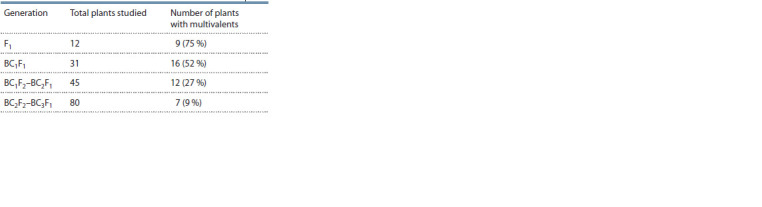
Results of the study of chromosome pairing in metaphase I of meiosis of generation F1 and BC1F1–BC3F1

In general, the percentage of plants with multivalents did not differ by crossing combinations.

**Fig. 1.**
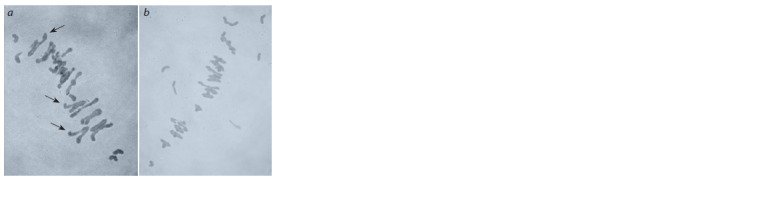
Chromosome pairing in metaphase I of meiosis in RS5 × Krasnodarskaya 99 hybrid plants: а, BC1 (14II + 4I + 2III + 1IV); b, BC2 (19II + 4I). Multivalents are indicated by arrows.

A large number of multivalents (75 %) was observed in F1 plants obtained from crossing the recombinant RS5 form with common wheat, which is explained by the direct influence of the S genome chromosomes, which are a part of the recombinant sterile form, on the pairing of different genomes chromosomes. Further, along with the increasing number of backcrosses, which were also carried out in order to overcome the low fertility of F1 hybrid plants, the number of plants with multivalents significantly decreases (up to 9 %). Examples of chromosome pairing in metaphase I of meiosis in hybrid plants are shown in Fig. 1.

The selection of plants for fertility and disease resistance, self-pollination contributed to the meiosis stabilization and necessary signs consolidation. As a result of the plants selection by the chromosomes number close to common wheat (42), 82 lines have now been obtained from the population of hybrid plants obtained on the basis of RS5 synthetics. This article presents the results of studying 12 lines that are closest to the recipient varieties according to the phenotype.

When using the RS5 form, the main purpose was the transmission of common wheat disease resistance. In this regard, an assessment of lines was fulfilled for the most common and harmful diseases – leaf rust (Puccinia triticina Eriks.), yellow rust (Puccinia striiformis f. sр. tritici) and powdery mildew (Blumeria graminis f. sр. tritici). Characterization of introgression lines RS5 × T. aestivum for disease resistance for 2019–2021 is given in Table 2.

**Table 2.**
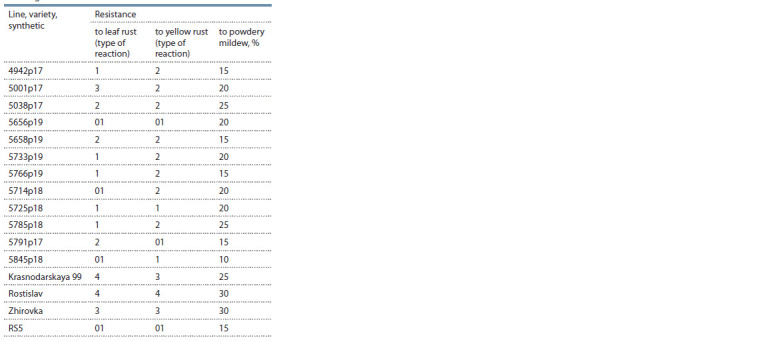
Disease resistance of introgression lines RS5 × T. aestivum for 2019–2021

Eleven lines were resistant to leaf rust. Eight lines showed high resistance with reaction type 01 and 1: 4942p17, 5656p19, 5733p19, 5766p19, 5714p18, 5725p18, 5785p18 and 5845p18. The line 5001p17 was susceptible. The remaining lines had moderate resistance to this disease.

Resistance to yellow rust was carried by all 12 lines, 4 of which, 5656p19, 5725p18, 5791p17 and 5845p18 have the type of reaction to infection 01 and 1.

Resistance to powdery mildew was shown by 10 lines, with the exception of lines 5038p17 and 5785p18.

Of particular value for breeding are lines that are resistant to a complex of diseases. Three lines, 5001p17, 5038p17 and 5785p18, had group resistance to two and nine lines to all three diseases. The 5845p18 line had high resistance to all three diseases. The diversity of disease resistance lines may indicate different introgressions of foreign genetic material into the genome of common wheat.

In order to determine the form of the transferred material from the synthetic RS5 form, the studied lines were crossed with one of the most meiotically stable varieties of common wheat Krasnodarskaya 99 and meiosis was studied in hybrid F1 plants (Table 3).

**Table 3.**
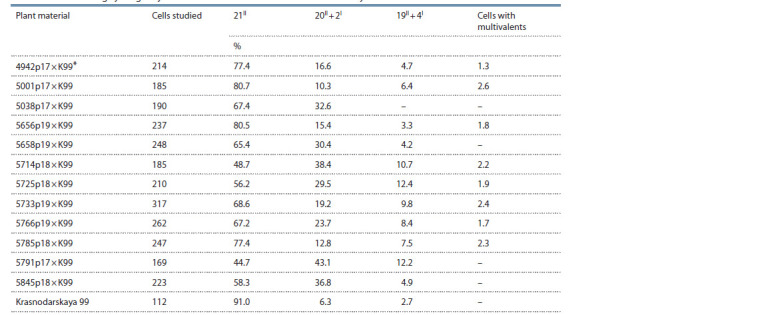
Analysis of meiosis in metaphase I in maternal pollen cells F1 hybrids obtained from crossing cytologically stable RS5 × T. aestivum lines with Krasnodarskaya 99 * Hereinafter: K99 is a variety of wheat Krasnodarskaya 99.

The association of chromosomes of hybrid plants F1 20II + 2I and 19II + 4I may indicate the substitution of one or two pairs of wheat chromosomes with foreign ones. Such substitutions can occur in 4 lines out of 12 analyzed – 5038p17, 5658p19, 5791p17 and 5845p18. The hybrids of Krasnodarskaya 99 with the other lines have the presence of multivalents, which indicates that they can carry translocations from the RS5 synthetic, Rostislav and Zhirovka varieties. Hybrid plants of the lines 5714p18, 5725p18, 5733p19, 5766p19 along with multivalents form a significant number of cells (about 30 %) with the association of chromosomes 20II + 2I and 19II + 4I. Probably, both translocations and substituted chromosomes may be present in these lines.

To identify the genetic material from the RS5 synthetic and changes in the genome of the obtained lines, the C-banding method was used. Of the eight analyzed lines, six revealed transfer from RS5 synthetics (Table 4).

**Table 4.**
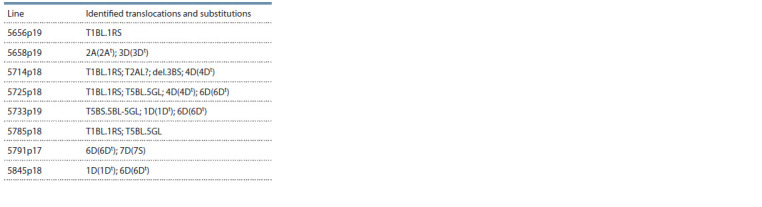
Results of the analysis RS5 × T. aestivum introgression lines by C-banding

**Fig. 2.**
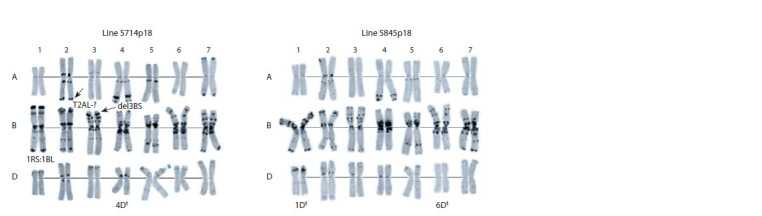
Karyotypes of introgression lines 5714p18 and 5845p18 with genetic material of the recombinant synthetic form RS5.

The rearrangements mainly affected the chromosomes of the D genome. In most cases, the lines carry substituted chromosomes from Ae. tauschii. The most common rearrangements affect chromosomes 1D, 4D and 6D (Fig. 2).

Substitutions 2A(2At) and 3D(3Dt) were identified in line 5658p19. The line 5791p17 has a 6D chromosome substitution from Ae. tauschii and 7D from Ae. speltoides. It should be noted that introgression lines with chromosomal substitutions 4D(4Dt), 6D(6Dt) from Ae. tauschii and 7D(7S) from Ae. speltoides were obtained for the first time. Translocation T1BL.1RS from the recipient cultivar Rostislav was revealed in line 5656p19. Translocation T5BL.5GL obtained from the recipient cultivar Zhirovka is present in three lines – 5725p18, 5733p19 and 5785p18. The obtained introgressive lines are of particular interest as possible new disease resistance genes donors, in particular, to leaf rust, transferred from the species Ae. tauschii and Ae. speltoides. Currently, 5 resistance genes from Ae. tauschii: Lr21, Lr22a, Lr32, Lr39, Lr42 and 6 resistance genes transmitted from Ae. speltoides: Lr28, Lr35, Lr36, Lr47, Lr51, Lr66 (McIntosh et al., 2015) are added to the catalog of wheat gene symbols. DNA markers were used to identify genes for resistance to leaf rust. Earlier (Davoyan E.R. et al., 2012, 2018), we analyzed the synthetic forms Avrodes and M.it./Ae. tauschii for the presence of effective leaf rust resistance genes Lr28, Lr35, Lr47, Lr51 from Ae. speltoides and Lr39 from Ae. tauschii. The resistance gene Lr36 was not included in the analysis due to the lack of an effective molecular marker for it. Identification of the Lr66 gene was not performed at this stage. It was found that the synthetic form of Avrodes has only Lr28, Lr35 and Lr51 of the listed genes, and the synthetic form M.it/Ae. tauschii has the Lr39 gene. Based on this, the obtained introgressive lines were analyzed only for the presence of effective leaf rust resistance genes Lr28, Lr35, Lr39 and Lr51. The presence of the desired genes has not been established in any of the 12 lines.

To determine the prospects for involving the obtained lines in breeding practice, they were evaluated according to the technological qualities of grain and productivity components. This paper presents the results of six most phenotypically interesting lines evaluation of the 2019 harvest.

One of the most important agronomic traits, especially for lines carrying alien genetic material, is the technological characteristics of grain and flour. Alien introgression can significantly affect the technological qualities of grain and flour. The results of the analysis of lines for some technological parameters are presented in Table 5. The protein and gluten content of the lines largely depend on the conditions of the growing season. All studied lines exceeded the best recipient cultivar Krasnodarskaya 99 in terms of protein and gluten content. The lines 5656p19 and 5725p18 had the highest levels – 18.6 and 17.9 % protein, 36.8 and 37.0 % gluten, respectively. The protein and gluten content of the Krasnodarskaya 99 variety was 14.4 and 26 % (see Table 5).

**Table 5.**
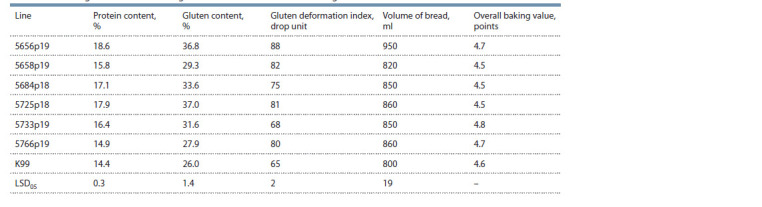
Technological characteristics of grain and flour RS5 × T. aestivum introgression lines of common wheat

Grain technological characteristics are determined by the protein and gluten content, as well as the qualitative indicators of gluten, which, in turn, determine such important characteristics as bread volume, crumb color, bread taste characteristics, etc. As a rule, the lines with alien genetic material have deterioration in the gluten quality. Thus, all the analyzed lines have high levels of gluten deformation index compared to the recipient variety Krasnodarskaya 99. However, the lines 5684p18 and 5733p19 had the gluten quality corresponding to group I according to State Standard, and the lines 5656p19, 5658p19, 5725p18 and 5766p19 had quality group II according to State Standard, which is a good indicator in general for introgressive lines. The volume output of bread in two lines 5658p19 and 5656p19 was 820 and 950 ml, respectively, exceeding the volume of bread of the recipient variety Krasnodarskaya 99 (800 ml). There were significant differences between the lines according to the indicator of the general baking assessment. Three lines: 5658p19, 5684p18 and 5725p18 (4.5 points) were inferior in this indicator to the recipient variety Krasnodarskaya 99 (4.6 points), and two lines, 5656p19 and 5766p19, having a score of 4.7 points, slightly exceeded the indicator of the Krasnodarskaya 99 variety. The line 5733p19 had the best baking rating out of all the lines – 4.8 points.

To study productivity, the following characteristics were used: the weight of 1000 grains, the weight of the grain, and the number of spikes per square meter (Table 6). The weight of 1000 grains in the lines varied from 38.0 (5733p19) to 43.9 g in line 5766p19, with an average value of the Krasnodarskaya 99 variety – 37.4 g. All lines, with the exception of 5733p19, significantly exceed the Krasnodarskaya 99 variety in this sign. Lines 5658p19, 5684p18 and 5766p19 form a smaller number of spikes per 1 m2. In the other three lines, the differences from the Krasnodarskaya 99 variety were insignificant. The highest yield (600.3 g/m2), comparable to the Krasnodarskaya 99 variety (603.7 g/m2), had the line 5766p19. The other lines were significantly inferior to the Krasnodarskaya 99 variety.

**Table 6.**
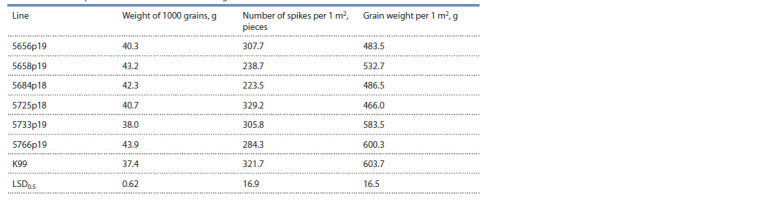
Yield components of RS5 × T. aestivum introgression lines

## Discussion

The creation and use of the synthetic form of RS5 was primarily associated with the possibility to transfer new introgressions from Ae. tauschii and Ae. speltoides to common wheat and, as a result, new disease resistance genes. Along with the selection of stable hybrid plants, their cytological study is important. The study of chromosome pairing in metaphase I of meiosis in RS5 × T. aestivum hybrid plants revealed a relatively large number of plants with multivalents in the early generations of F1 and BC1F1 – 75 and 52 %, respectively. Such results are due to the ability of the synthetic form of Avrodes, obtained with the participation of Ae. speltoides, to cause homeologous pairing of chromosomes (Tsatsenco et al., 1993). A significant decrease in the number of plants with multivalents in subsequent generations of BC2F1–BC3F1 (9 %) may be associated with the stabilization of the number of chromosomes and their association in meiosis towards common wheat, as well as a decrease in the genetic material Ae. speltoides in them.

The 12 RS5 × T. aestivum lines of the BC2F6–BC3F5 generation selected for the study differed in resistance to leaf rust, yellow rust and powdery mildew. Lines with the types of reaction to leaf rust 01, 1 and 2, to yellow rust 01, 1 and 2, with a degree of powdery mildew damage of 10, 15 and 20 % were identified. The lines differ in their resistance to the complex of these diseases as well. The diversity of disease resistance lines may indicate different transfers of the RS5 genetic material in the genome of common wheat and the possible transfer of a new resistance gene(s).

Cytological analysis (C-banding) revealed chromosomal rearrangements in 6 out of 8 studied lines. The rearrangements mainly affected the chromosomes of the D genome – 1D, 3D, 4D, 6D, and 7D. In most cases, the genetic material from the synthetic RS5 form in the studied lines was found to be presented in the form of substituted chromosomes from Ae. tauschii. In one line – 5791p17 the substitution of chromosomes 6D from Ae. tauschii and 7D from Ae. speltoides was identified. It should be noted that chromosomal substitutions 4D(4Dt), 6D(6Dt) from Ae. tauschii and 7D(7S) from Ae. speltoides were obtained for the first time. Active participation in rearrangements of chromosomes of the D genome is explained by the fact that, firstly, Ae. tauschii is a donor of the D genome, secondly, in the synthetic form of Avrodes (BBAASS), the D genome of common wheat is replaced by the S genome from Ae. speltoides. In line 5656p19, translocation T1BL.1RS from the recipient cultivar Rostislav was revealed. At the same time, in contrast to the Rostislav variety, this line is resistant to leaf rust (01) and yellow rust (01) and has high levels of protein and gluten (18.6 and 35.8 %, respectively). Probably, the transfer of genetic material from RS5 of this line occurred through recombination, which is not detected by the C-banding method. The T5BL.5GL translo- cation obtained from the recipient cultivar Zhirovka was found in three lines – 5725p18, 5733p19, and 5785p18. Currently, this translocation does not provide resistance to leaf rust, yellow rust and powdery mildew.

The genes of resistance to leaf rust Lr21, Lr22a, Lr32, Lr39, Lr42 from the species Ae. tauschii and Lr28, Lr35, Lr36, Lr47, Lr51, Lr66, LrASP5 from Ae. speltoides were transferred to common wheat (Adonina et al., 2012; McIntosh et al., 2015). These genes were transferred from Ae. tauschii to the wheat chromosomes 1D, 2D, 3D, 2D and 1D, respectively; from Ae. speltoides – to 4A, 2B, 6B, 7A, 1B, 3A and 5B, respectively (Friebe et al., 1996; Helguera al., 2000, 2005; Marais et al., 2010). Despite the rather large number of transferred genes, it is possible that other leaf rust resistance genes may be present in these species, which is also evidenced by the results obtained earlier (Davoyan R.O. et al., 2017).

Based on the marker analysis, it was previously assumed that the synthetic form of M.it/Ae. tauschii has Lr39 of the listed genes, while Avrodes has only three: Lr28, Lr35, and Lr51. The desired genes were not detected in any of the 12 analyzed lines. Probably, these lines may have new leaf rust resistance genes derived from Ae. tauschii and Ae. speltoides.

Genetic material of wild relatives in introgression lines of common wheat, along with positive traits, can also carry undesirable ones, such as lengthening the growing period, deterioration of baking qualities, lodging tendency, decreased yield, etc. (Knott, 1989; Brevis et al., 2008; Timonova et al., 2012; Leonova, Budashkina, 2016).

The study of the 6 most interesting lines by phenotype revealed their diversity in productivity and technological characteristics of grain and flour. The studied lines exceeded the recipient cultivar Krasnodarskaya 99 in protein and gluten content. The lines 5656p19 and 5725p18 had the highest indices – 18.6 and 17.9 % protein, 36.8 and 37.0 % gluten, respectively. Despite the fact that all the analyzed lines have high levels of gluten deformation index compared to the Krasnodarskaya 99 variety, they form gluten corresponding to the first and second groups of state standard and have either an equal with Krasnodarskaya 99, or a higher overall baking rating. Thus, along with disease resistance, the studied lines can be used as donors to improve the technological qualities of grain and flour.

All lines, with the exception of 5733p19, significantly exceeded the weight of 1000 grains of the Krasnodarskaya 99 variety. According to the number of spikes per 1 m2, the lines have either equal (5656p19, 5725p18, 5733p19) or lower indicators (5658p19, 5684p18 and 5766p19) compared to the Krasnodarskaya 99 variety. With the exception of the 5766p19 line, all the others were significantly inferior to the Krasnodarskaya 99 in terms of grain weight per 1 m2. Based on the obtained data, the reduced productivity of the lines compared to Krasnodarskaya 99 can be tentatively attributed to the fact that against the background of a significantly high protein content (with the exception of the 5766p19 line), which, as a rule, negatively correlates with yield, they form either an equal or significantly smaller number of spikes per 1 m2. It should also be noted that Krasnodarskaya 99 is one of the high-yielding varieties of winter common wheat.

## Conclusion

Thus, the obtained results indicate a wide variety of created introgression lines and the effectiveness of using the synthetic RS5 form for transferring genetic material from Ae. tauschii and Ae. speltoides to common wheat.

## Conflict of interest

The authors declare no conflict of interest.
